# A Rapid Micromixer for Centrifugal Microfluidic Platforms

**DOI:** 10.3390/mi7050089

**Published:** 2016-05-10

**Authors:** Ziliang Cai, Jiwen Xiang, Hualing Chen, Wanjun Wang

**Affiliations:** 1 Department of Mechanical and Industrial Engineering, Louisiana State University, Baton Rouge, LA 70803, USA; zcai4@lsu.edu (Z.C.); jxiang3@lsu.edu (J.X.); 2 College of Mechanical Engineering, Xi’an Jiaotong University, Xi’an 710049, China; hlchen@mail.xjtu.edu.cn

**Keywords:** micromixer, centrifugal microfluidics, PDMS, active micro-mixing, lab-on-CD, flyball governor actuated, chaotic mixing

## Abstract

This paper presents an innovative mixing technology for centrifugal microfluidic platforms actuated using a specially designed flyball governor. The multilayer microfluidic disc was fabricated using a polydimethylsiloxane (PDMS) replica molding process with a soft lithography technique. The operational principle is based on the interaction between the elastic covering membrane and an actuator pin installed on the flyball governor system. The flyball governor was used as the transducer to convert the rotary motion into a reciprocating linear motion of the pin pressing against the covering membrane of the mixer chamber. When the rotation speed of the microfluidic disc was periodically altered, the mixing chamber was compressed and released accordingly. In this way, enhanced active mixing can be achieved with much better efficiency in comparison with diffusive mixing.

## 1. Introduction

Micromixing is a critical process in miniaturized analysis systems. However, mixing of fluids at the microscale faces a big challenge because viscous effects dominate at small scales, where the flow is laminar, and the mixing between different streams in the flow mainly depends on the molecular diffusion. Unfortunately, the diffusive mixing is slow compared with the convective mixing; thus, the mixing length for molecular diffusion is always prohibitively long which negates most of the benefits of miniaturization. In recent years, to reduce the mixing time, many efficient chaotic micromixers have been explored. The concept of the chaotic mixer is to generate chaotic advection via stretching and folding fluids. Generally, micromixers can be broadly classified as two types: passive micromixers and active micromixers. In passive micromixers, the flow field is perturbed by changing the geometry of channels or adding geometric obstacles such as the square-wave micromixer [1], the zigzag micromixer [2], and the staggered herringbone micromixer [3]. In active micromixers, fluids are always perturbed by using an external energy source such as mechanical pulsation [4], acoustic vibration [5], magnetic force [6], electrohydrodynamic force [7] and electroosmotic force [8]. These reported strategies can improve mixing performance for conventional microfluidic systems to some extent.

In the past few decades, centrifugal microfluidic systems have emerged as an important branch of the microfluidic systems. The liquid mixing has also become a challenging issue for these types of micro systems. Because of the limitation of the spinning platform, the aformentioned approaches cannot be easily implemented for centrifugal microfluidic systems. First, the passive mixer usually requires high pressure due to the resistance in the fluidic channel. In the centrifugal microfluidics, the pressure exerted on the liquid generally can only be applied by the centrifugal force as the disc rotates. It is not practical to achieve the required pressure level in most cases. For the active mixers not based on centrifugal forces, the internal energy sources are difficult to integrate with centrifugal microfluidic platforms. Considerable effort therefore has been dedicated to the development of the micromixing technologies suitable for centrifugal microfluidic platforms. The most common method is known as “shake-mode mixing”, which is to continuously alter the rotating direction of the disc [9]. The action of the liquid due to the frequent reversal of rotational direction can improve the mixing effect. However, a consequence of shake-mode mixing is that the disk is momentarily stationary when the rotational direction is altered, which can possibly affect the operation of other units on the disc such as the priming process for the siphon valves. Some other methods have also been reported. Noroozi *et al.* [10] generated a reciprocating flow between two chambers of a polydimethylsiloxane (PDMS) chip. However, the mixing time is still too long. Kong *et al.* [11] blew compressed gas into the mixing chamber and agitated chaotic mixing. The limitations of this method are that the external gas may contaminate the sample liquid as well as the additional complexity of controlling the external gas.

In this paper, we report a micromixing technology for the centrifugal microfluidic platform. This technology is based on a flyball governor system described in our previous reported work [12,13]. The mixing chamber is periodically compressed simply by altering the spinning speed of the disc to enhance the mixing effect. The operation of this mixer is in a non-contact fashion without sample contamination issues and with no external energy source required.

## 2. Operational Principle and Design

**Figure 1:**
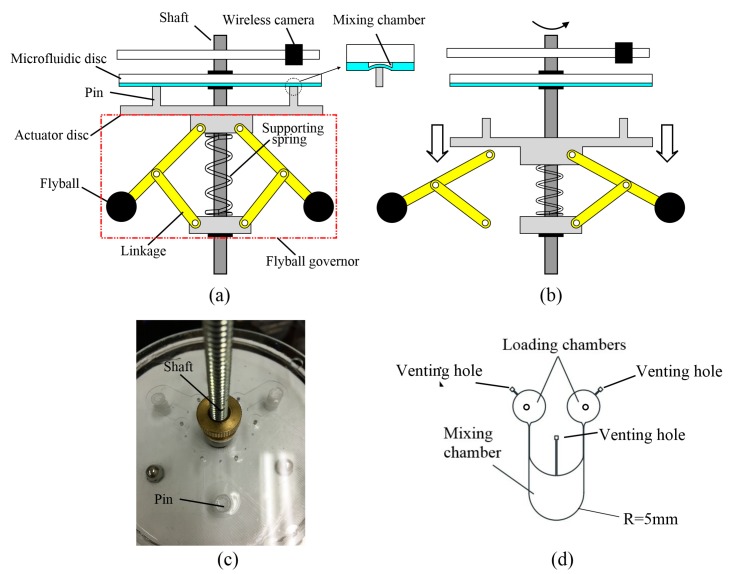
(**a**) Schematic diagrams of the mixing system. It consists of three parts from top to bottom: the microfluidic disc, actuation disc, and the flyball governor system. The mixing chamber is in the compressed state initially. (**b**) The actuation disc was pulled down by the flyball governor as the spinning rate of the disc increases. At the same time, the pin loses contact with the covering membrane of the mixer chamber. (**c**) Close-in view of the mixer, the PDMS layer consists of three identical patterns. The microchannel is 300 μm high and 300 μm in width. (**d**) Schematic design of a single microfluidic system used for testing the performance of the mixer.

The operation of the mixing technique is based on actuation using a fly-ball governor system [12,13,14]. Figure 1a shows the schematic diagram of an assembled platform. The system consists of three major parts: the flyball governor, the actuation disc with the pins, and the microfluidic disc. The system is equipped with a brushless motor with controller (BLDC-38S, Zhengke Motor Co., Wenzhou, China) to spin the disc through the coupling joint. An LED tachometer was installed to monitor the rotating speed. The speed of the motor is controlled by pulse width modulation. The flyball governor comes with three identical flyballs, whose weight can be adjusted conveniently. A metal tube was placed in the center of the actuator disc, which is fixed on the top of flyball governor, so that it can slide up and down freely along the shaft. The actuator disc was fabricated by 3D printing to avoid assembly and minimize the weight of the rotary system. Three pins were printed on the actuator disc and placed under the mixing chamber after assembly. The micoflufidic disc is mounted on the top the system, above the actuator disc. It consists of a PDMS layer and a polymethyl methacrylate (PMMA) substrate. The mixing chamber is enclosed in the PDMS layer. The fabrication of microfluidic disc is completed by standard photolithography and PDMS casting. When the system is in stationary state, the supporting spring pushes the actuation disc up and the pin is used to press the covering membrane of the mixing chamber. As the system rotates, the centrifugal force helps to overcome the force of supporting spring in the center of the flyball governor, and pulls the actuation disc to move downward, and the pin therefore loses the contact with the covering membrane of the mixing chamber as shown in Figure 1b. Figure 1c shows a photograph of the prototype system with three sets of the identical mixers designed in a symmetric fashion. In the photo, the dark circles are the images of the pins beneath the transparent fluidic platform. The chaotic mixing is generated in a cyclic compressing-releasing process by controlling the spinning rate of the disc. Figure 1d shows the schematic design of the prototype mixer system with two loading chambers and one mixing chamber. The venting hole in Figure 1d was needed to release air so that sample fluids can be supplied to the mixing chamber. The flow channel between the venting hole and the mixing chamber was designed to be small enough to permit air to flow through while the liquid samples are blocked because of their higher viscosity and the resulting higher surface tension force.

**Figure 2:**
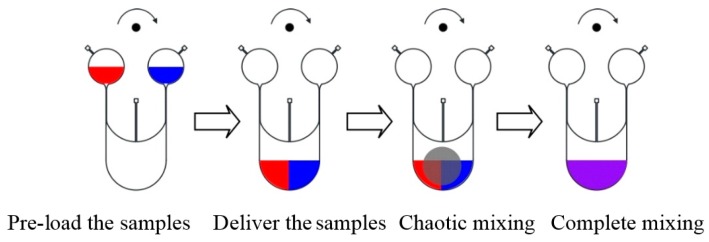
Operational principles of the chaotic mixing system. The two different colors represent two fluid samples that are introduced into the mixing chamber first. The dark circle in the 3rd figure indicates the pin pressing against the covering membrane of the mixing chamber.

Figure 2 schematically demonstrated the operational principle of the micro-mixer. The mixing process is accomplished in three phases:

(1) Pre-load the samples. At the initial state, the sample liquids are introduced to the loading chambers.
(2) Deliver the sample liquids to the mixing chamber by increasing the spinning rate of the disc. The vent hole in the figure is needed to release the air as the fluid sample flow into the mixing chamber and pushes air out.
(3) Chaotic mixing. After samples are introduced into the mixing chamber, the rotational speed of the system is oscillated to drive the actuation disc to move up and down. The pin on in the actuation disc is therefore pressed against the covering membrane of the mixing chamber and then released repeatedly. The fluids in the mixing chamber are therefore agitated to cause chaotic mixing.

## 3. Experimental Results and Discussions

### 3.1. Fabrication of the Microfluidic Disc

**Figure 3:**
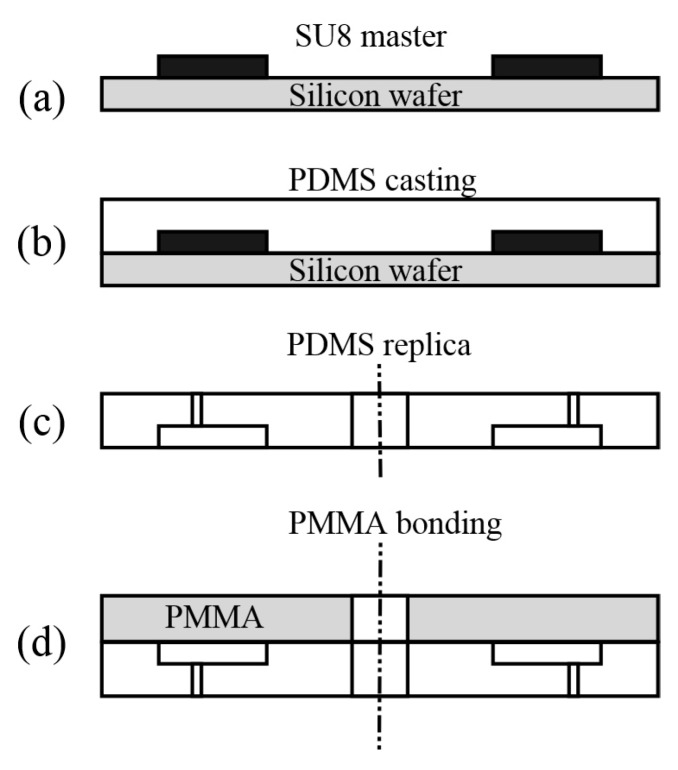
The fabrication process for the microfluidic disc. (**a**) SU-8 photolithography and development; (**b**) Pouring of PDMS and curing; (**c**) Demolding of PDMS replica and punching holes; (**d**) Bonding PDMS to PMMA disc.

The microfluidic disc was fabricated in four steps as shown in Figure 3. The first step was to fabricate the SU-8 master mold with soft lithography method. A thick layer of SU-8 100 photoresist (MicroChem, Westborough, MA, USA) was first spin-coated on a four-inch silicon wafer. Next, fluidic patterns on a mask were transferred to the photoresist in UV exposure followed by the soft bake of photoresist. After the postbake and development, the master mold was obtained for the PDMS casting fabrication step. A mixture of PDMS (Sylgard184, Dow Corning, Midland, MI, USA) was prepared by mixing the base and curing agent in a 10:1 (*m*/*m*) ratio and casting it on the master mold. After being cured at 85 °C in the oven for 1 h, the PDMS layer was peeled off the master mold and the holes were punched using a sharpened blunt needle. The top and bottom PDMS layers were obtained through similar procedures.

In this study, a 4-inch PMMA disc was used as the substrate. The central hole, through which the shaft of the motor passes, and connection holes were drilled by computer numerical control (CNC) machinery. After the PDMS layers were fabricated, the three parts were aligned and directly bonded together [14]. The bonded components were gently pressed to eliminate air bubbles. The reversible bond was performed without any chemical or plasma treatment. The experiments showed that the direct bonding between the PDMS and PMMA was strong enough to fulfill the requirements of our experiment and no leakage was observed during the operation. Two different sizes of pins were designed, one at 4.2 and 8 mm, respectively.

### 3.2. Test of the Prototype Micromixer

**Figure 4:**
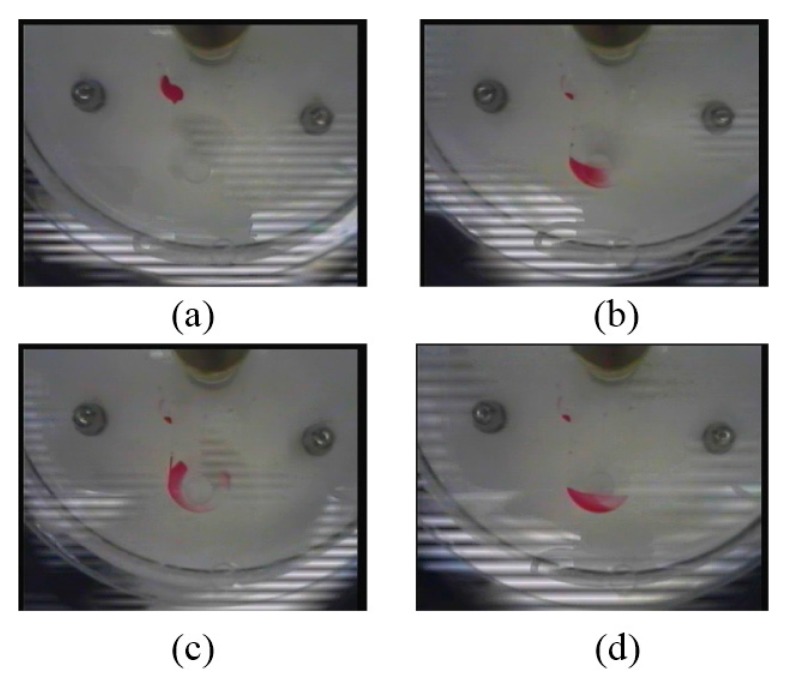
Photo images showing one complete cycle of mixing operation on the centrifugal microfluidic platform: (**a**) the samples were introduced into the loading chambers; (**b**) the samples were delivered to the mixing chamber; (**c**) the mixing chamber was compressed by the pin connected to the flyball governor at low mixing frequency; and (**d**) the pin was released at higher spinning speed.

Experiments were conducted to test the mixing efficiency of the chaotic micromixer based on flyball governor actuation using a pin size of 4.2 mm. Photo images in Figure 4 demonstrated four major steps of a mixing cycle. First, samples of 50 μL of red dye solution and 50 μL of deionized (DI) water were introduced into the loading chambers as shown in Figure 4a, with the red dye on the left chamber and the DI water on the right chamber, respectively. The mixing chamber was initially compressed. The disc was first spun at 800 rpm to remove the compression on the mixing chamber and propel the liquids into the mixing chamber as shown in Figure 4b. Next, the spinning speed decreased to 600 rpm so that the pin pressed the chamber again to cause rapid mixing as shown in Figure 4c. When the pin was driven away, it is clear that the liquids were better mixed in Figure 4d. For the experimental system, it was found that 600 rpm was an appropriate speed. It was found that, at this speed, the pin is about to fully compress the chamber. With the speed significantly higher than 600 rpm, the compression effect became too weak. If the speed is significantly lower than 600 rpm, the acceleration process was increased accordingly and the mixing time was also increased.

**Figure 5:**
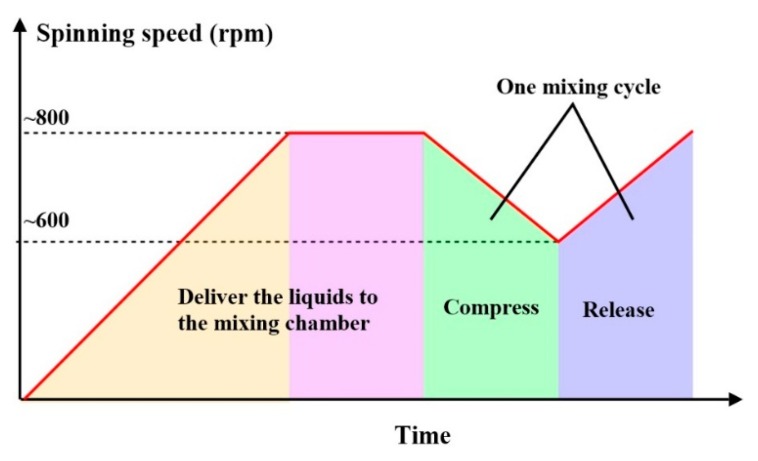
The spinning speed profile followed in the experiments of the mixer. The compressing-releasing times are controlled to be equal.

Figure 5 shows the corresponding sequence of spinning speed variation during each cycle of the mixing operation. The cycle is repeated until the liquids are completely mixed.

**Figure 6:**
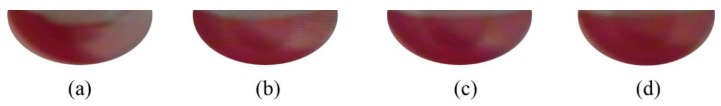
Images of the liquids after (**a**) one mixing cycle, (**b**) two mixing cycles, (**c**) three mixing cycles, and (**d**) four mixing cycles, respectively.

As shown in Figure 1, a wireless camera is installed above the microfluidic disc and rotates with it. The camera is used to monitor the movements of the samples and transmit the digital images wirelessly to a computer for data processing and display. Figure 6 gives the sequential images obtained by the wireless camera. These images demonstrated the effectiveness of the mixing after one cycle, two cycles, three cycles, and four mixing cycles, respectively. The total time for completing four mixing cycles was 15 s.

**Figure 7:**
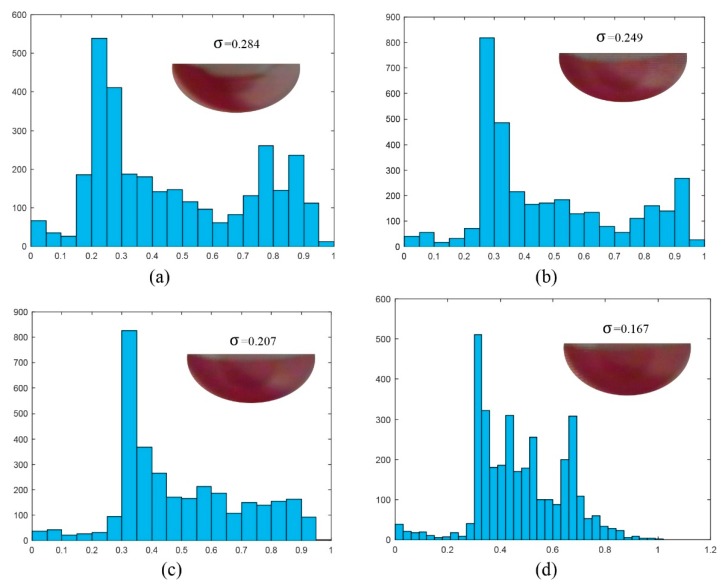
Histograms and standard deviations obtained from the gray intensities of the photo images of the mixing chamber after (**a**) one mixing cycle, (**b**) two mixing cycles, (**c**) three mixing cycles, and (**d**) four mixing cycles, respectively.

To quantify effectiveness of the mixing system, the standard derivation of the pixel intensity of the image of the fluid sample in the mixing chamber was used to numerically represent the state of the mixing process. After each cycle, the image of mixer chamber was captured and stored. For each image, the area of the image for the mixing chamber was digitally cut out as the region of interest. These images were then imported into Matlab for further processing. The gray intensities for all the pixels were obtained. The standard derivation for each image was then calculated. A smaller standard derivation stands for better mixing effect. Figure 7 shows the histograms and the standard derivations after one, two, three, and four cycles, respectively. As the number of pressing-releasing mixing cycles increased from one to four, the gray-scale distributions of the images became more uniform and the standard deviation of the images dropped from 0.284 to 0.167. It also can be noted that, as mixing continues, the distribution of pixel intensities shifts towards unity in the histograms. It also can be noted that using the image analysis method to estimate mixing effective is limited by the camera resolution and the irregular shape of the liquid sample in the mixing chamber. However, the standard deviation of the gray intensities still provides a useful estimation of the mixing performance.

**Figure 8:**
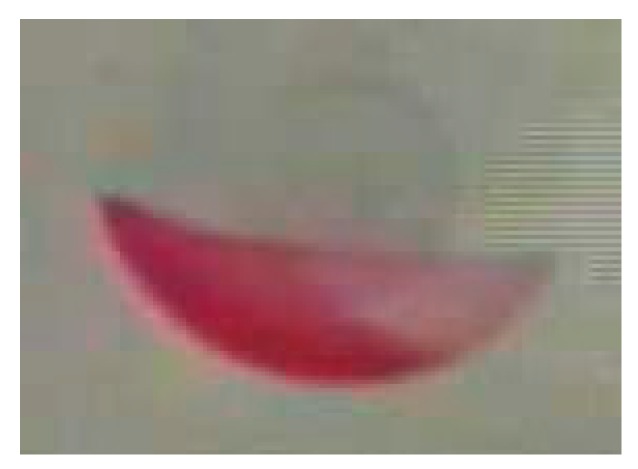
Image of the mixing chamber after the disc was spun after 15 s without using the compression-releasing mechanism. The standard derivation was calculated to be 0.25.

For the sake of comparison, experiments were also conducted for the mixing effect of pure diffusion for the same centrifugal platform without using the compressing-releasing mechanism. The same amounts of sample fluids were transferred into the mixing chamber and spun with the disc for 15 s. Because of no compression-releasing actuation, the mixing is therefore primarily based on diffusion under the centrifugal environment. The photo image in Figure 8 shows the mixing result. As can be observed, the liquids stayed mostly unmixed. The standard deviation is calculated to be 0.25. By comparison, the standard deviation of the mixing results shown in Figure 6d after 15 s was at 0.167, which is significantly better.

A study was also conducted to investigate the effect of the operational parameters on the mixing results. First, different rotational speeds of disc were tested. The experimental results showed that the pin started to lose contact with the covering membrane of the mixer to perform the “release” operation at around 800 rpm. Higher rotating speed was not helpful to deliver better mixing performance due to the extra time needed for longer deceleration process in the “compress” operation. Therefore, the highest rotational speed of disc was set to be at 800 rpm.

**Figure 9:**
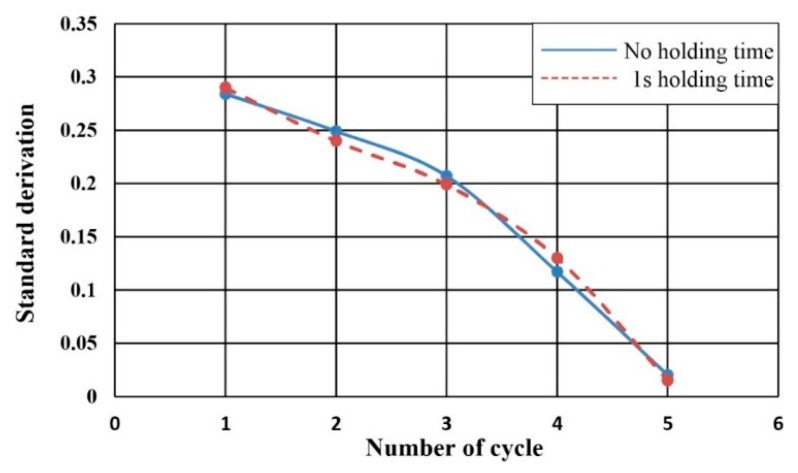
Comparison of the standard deviation of the mixing performance with and without the holding phase.

To study the effect of the rotation pattern of the disc on the mixing efficiency, an extra “holding phase” was added in the compression-release mixing process. In this approach, the compression by the pin on the mixing chamber was maintained for a short period (1 s) and then released instead of repeating the fast compression-release process as presented in Figure 5. Standard deviations of the mixing images for active mixing with one second of “holding phases” after one, two, three, four, and five cycles were obtained, and the results are presented in Figure 9. As can be obtained from the results in Figure 9, the extra holding phase did not improve the mixing effectiveness. In the holding phase, the mixing only relies on diffusion. These experimental results further confirmed that the improvement of the mixing effect was primarily caused by the chaotic mixing mechanism rather than the diffusive one.

**Figure 10:**
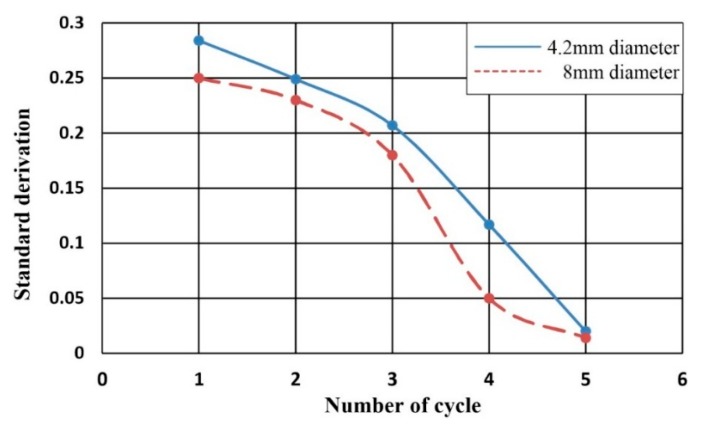
The standard deviation of the mixing performance with two different pin sizes.

For design purposes, the effect of the pin size was also studied. An 8 mm pin was used to replace the 4.2 mm pin used in the previous experiments. The comparison of the results is demonstrated in Figure 10. It can be observed that the mixing performance was improved. The main reason is that the larger pin can produce a stronger compression effect to boost the chaotic mixing. However, the size of the pin is limited by the size of the mixer chamber. As a result, to choose a pin size not much smaller than that of the mixer chamber is therefore preferred.

This mixing technique works on the chaotic mixing caused by the external mechanical system, and the primary advantage of this mixing method is that it does not require extra entities integrated into the disc such as beads or magnets. The technique can effectively avoid any possible contact contamination of the samples because the actuation of the mixer was achieved on the outside of the covering membrane. Additionally, this mixing technique also relies on the function of the flyball governor for the pinch-valves and inward-pumping technologies reported in our earlier work [12,13]. It is therefore quite easy to integrate the mixer with the pinch-valving and inward pumping units to achieve complex fluid handling on a centrifugal microfluidic platform based on flyball governor actuation. One possible drawback of the mixing method is its applications in dealing with cells or beads may be limited because of possible damage during the compression process. Nevertheless, this mixing method can provide an efficient, non-contact way of mixing liquids on centrifugal microfluidic platforms, adding another possible alternative for the existing mixing technologies.

## 4. Conclusions

In this paper, the application of the flyball governor in the micro-mixing function on a centrifugal platform was explored. By controlling the spinning speed of the flyball governor to fluctuate periodically, the sample fluids in the micromixer were chaotically mixed by the repeated compression and releasing actions of the pin pressing against the covering membrane of the mixer chamber. By visual inspection and digital analysis of the photo images of the fluid sample in the mixer chamber to compare the standard deviations of the gray intensities, it was confirmed that this chaotic mixing method enables effective mixing of liquids in 15 s. The technology can be used alone or integrated with other devices such as micropump and pinch-valves actuated using the same flyball governor. It therefore bears significant potential for more complicated lab-on-CD applications.

## References

[1] Liu R.H., Stremler M.A., Sharp K.V., Olsen M.G., Santiago J.G., Adrian R.J., Aref H., Beebe D.J. (2000). Passive mixing in a three-dimensional serpentine microchannel. J. Microelectromech. Syst..

[2] Mengeaud V., Josserand J., Girault H.H. (2002). Mixing processes in a zigzag microchannel: Finite element simulations and optical study. Anal. Chem..

[3] Stroock A.D., Dertinger S.K., Ajdari A., Mezić I., Stone H.A., Whitesides G.M. (2002). Chaotic mixer for microchannels. Science.

[4] Bottausci F., Mezic I., Meinhart C.D., Cardonne C. (2004). Mixing in the shear superposition micromixer: Three-dimensional analysis. Philos. Trans. R. Soc. Lond. A Math. Phys. Eng. Sci..

[5] Yang Z., Goto H., Matsumoto M., Maeda R. (2000). Active micromixer for microfluidic systems using lead-zirconate-titanate (PZT)-generated ultrasonic vibration. Electrophoresis.

[6] Mensing G.A., Pearce T.M., Graham M.D., Beebe D.J. (2004). An externally driven magnetic microstirrer. Philos. Trans. R. Soc. Lond. A Math. Phys. Eng. Sci..

[7] El Moctar A.O., Aubry N., Batton J. (2003). Electro-hydrodynamic micro-fluidic mixer. Lab Chip.

[8] Biddiss E., Erickson D., Li D. (2004). Heterogeneous surface charge enhanced micromixing for electrokinetic flows. Anal. Chem..

[9] Grumann M., Geipel A., Riegger L., Zengerle R., Ducree J. (2005). Batch-mode mixing on centrifugal microfluidic platforms. Lab Chip.

[10] Noroozi Z., Kido H., Micic M., Pan H., Bartolome C., Princevac M., Zoval J., Madou M. (2009). Reciprocating flow-based centrifugal microfluidics mixer. Rev. Sci. Instrum..

[11] Kong M.C.R., Salin E.D. (2012). Micromixing by pneumatic agitation on continually rotating centrifugal microfluidic platforms. Microfluid. Nanofluid..

[12] Cai Z., Xiang J., Wang W. (2015). A pinch-valve for centrifugal microfluidic platforms and its application in sequential valving operation and plasma extraction. Sens. Actuators B Chem..

[13] Cai Z., Xiang J., Zhang B., Wang W. (2015). A magnetically actuated valve for centrifugal microfluidic applications. Sens. Actuators B Chem..

[14] Cai Z., Xiang J., Chen H., Wang W. (2015). Membrane-based valves and inward-pumping system for centrifugal microfluidic platforms. Sens. Actuators B Chem..

